# Complement evasion by apicomplexans: Convergent strategies across diverse parasites

**DOI:** 10.1371/journal.ppat.1014435

**Published:** 2026-07-17

**Authors:** Sofía Espinosa-Hernández, Carlos J. Ramírez-Flores

**Affiliations:** Department of Infectomics and Molecular Pathogenesis, Center for Research and Advanced Studies (Cinvestav), Mexico City, Mexico; Childrens Hospital of Philadelphia, UNITED STATES OF AMERICA

## Abstract

Apicomplexan parasites encounter the complement system during extracellular stages of infection, where it imposes a major barrier to survival. Complement activation promotes pathogen elimination through opsonization, inflammation, and membrane attack complex (MAC) formation. To persist, apicomplexans deploy mechanistically distinct but functionally convergent strategies that modulate complement activity instead of fully inhibiting it. These include rapid invasion to limit extracellular exposure, recruitment of host complement regulatory proteins, surface shielding to prevent MAC insertion, and direct interference with complement activation. A central feature of these strategies is the regulation of C3 deposition and processing, which determines downstream effector outcomes. Here, we define a unifying framework for complement evasion across apicomplexans based on these four mechanisms.

## Introduction

The complement system is a central component of innate immunity that provides immediate defense against extracellular pathogens. Apicomplexan parasites encounter complement during transient extracellular stages, including egress, dissemination, and host cell invasion, where they are directly exposed to soluble complement components [1].

The magnitude and duration of this exposure differ across species. *Toxoplasma gondii* exhibits brief extracellular phases and is largely resistant to complement-mediated lysis, with complement acting primarily through opsonization and immune modulation [2]. At the other extreme, *Plasmodium falciparum* blood-stage parasites remain extracellular within the circulation for extended periods and are subject to sustained complement pressure [3]. These differences impose distinct selective constraints but converge on a shared requirement: tight regulation of complement activation [4].

## How does complement function as a central effector of innate immunity?

Complement activation is initiated through classical, lectin, and alternative pathways, all of which converge on the cleavage of C3 into C3a and C3b. C3b contains a reactive thioester that enables covalent attachment to target surfaces, forming the basis of opsonization. Surface-bound C3b associates with factor B and factor D to form the alternative pathway C3 convertase, which amplifies complement activation through a positive feedback loop [5].

This amplification is tightly regulated by host proteins. Factor H binds C3b and accelerates convertase decay while acting as a cofactor for factor I-mediated cleavage of C3b into inactive fragments. Formation of the C5 convertase enables downstream assembly of the membrane attack complex (MAC), initiated by C5b binding to C6 and C7, followed by insertion into membranes, recruitment of C8, and polymerization of C9 to form a lytic pore [5]. In parallel, the anaphylatoxins C3a and C5a promote chemotaxis, vascular permeability, and activation of innate immune cells. Regulation of C3 deposition and stability, therefore, determines the magnitude of complement effector activity [5].

Complement-derived signals may facilitate parasite dissemination by promoting activation of myeloid cells that serve as host cells for intracellular parasites such as *Toxoplasma gondii*. By restricting terminal pathway activity while preserving upstream signaling, apicomplexans support persistence and transmission.

## How do apicomplexans interact with the host’s first line of defense?

During early infection, apicomplexans encounter innate immune cells including neutrophils, monocytes, and dendritic cells, which contribute to early pathogen recognition and inflammatory signaling, followed by activation of innate lymphoid cells and natural killer cells that further amplify the immune response [6]. These cells detect parasites through pattern recognition receptors, including Toll-like receptors, which recognize conserved pathogen-associated molecular patterns and initiate inflammatory signaling [7].

Complement operates within this network as a central humoral effector that both amplifies and integrates innate immune responses. C3b deposition promotes phagocytosis through complement receptors, while the anaphylatoxins C3a and C5a enhance inflammatory signaling, recruit immune cells, and reinforce cellular activation [5]. In addition, complement can contribute to the activation of downstream immune responses. In apicomplexan infections, these responses are frequently associated with production of interferon gamma (IFN-γ), interleukin-12 (IL-12), and tumor necrosis factor alpha (TNF-α), which together promote macrophage activation and intracellular parasite control. These pathways enhance nitric oxide-dependent effector mechanisms that restrict parasite replication within host cells [8]. Complement-derived signals, therefore, link early extracellular recognition to intracellular immune responses that shape infection outcomes.

The contribution of complement varies across apicomplexans. *T. gondii* relies primarily on rapid invasion to limit extracellular exposure, whereas *P. falciparum* must actively regulate complement during prolonged circulation stages [9,10]. *Cryptosporidium* occupies an epicellular niche at the intestinal epithelium, potentially restricting access to soluble complement components [11], whereas *Babesia* encounters complement during erythrocyte invasion cycles [12]. These differences reflect parasite-specific biology and influence the relative importance of complement evasion mechanisms.

Consistent with this diversity, evidence from other apicomplexans supports a broader role for complement in host-parasite interactions. *Cryptosporidium parvum* binds and activates complement, with altered infection outcomes in complement-deficient models [13]. *Eimeria tenella* sporozoites are susceptible to complement-mediated neutralization via alternative and classical pathways [14]. In *Babesia*, complement contributes to erythrocyte invasion and antibody-dependent responses [15]. However, the molecular basis of complement evasion in these parasites remains poorly defined.

In parallel, apicomplexan parasites deploy additional immune evasion strategies, including modulation of cytokine signaling and secretion of immunomodulatory effectors, further supporting intracellular persistence.

## How do apicomplexan parasites evade complement-mediated attack?

### (i) Rapid escape from complement through host cell invasion

Apicomplexans minimize complement exposure by rapidly invading host cells through the coordinated activity of the apical complex. Microneme proteins mediate initial attachment and receptor engagement, while rhoptry neck proteins establish the moving junction, a ring-like structure that anchors the parasite to the host membrane and enables active penetration [16]. While these mechanisms are best characterized in *Plasmodium* and *Toxoplasma*, the apical complex and its core secretory functions are conserved across Apicomplexa, although their organization and contribution to host interaction vary among species.

The moving junction functions as a molecular sieve that excludes host transmembrane proteins, including immune receptors, from the site of entry, thereby limiting access of complement components and restricting C3b deposition at the parasite-host interface. Secretion of rhoptry contents also contributes to the formation of the parasitophorous vacuole, which rapidly separates the parasite from the extracellular environment [16].

Although the apical complex is broadly conserved across apicomplexans, the dynamics and cellular context of invasion differ substantially. *Plasmodium* invades erythrocytes through a multi-step process, whereas *Cryptosporidium* exhibits a modified invasion strategy associated with its epicellular niche [11,17].

These processes restrict the time window available for complement activation. *T. gondii* invades host cells within seconds, effectively preventing substantial C3 deposition. In contrast, *Plasmodium* merozoites remain extracellular for longer periods, increasing susceptibility to complement and necessitating additional evasion strategies.

### (ii) Recruitment of host complement regulatory proteins

In well-characterized apicomplexans, complement activation is suppressed through recruitment of host regulatory proteins to the parasite surface. Factor H binds C3b and accelerates decay of the alternative pathway C3 convertase while acting as a cofactor for factor I-mediated cleavage of C3b into inactive fragments. Similarly, C4b-binding protein regulates the classical pathway, and C1 esterase inhibitor blocks initiation [18].

**Fig 1:**
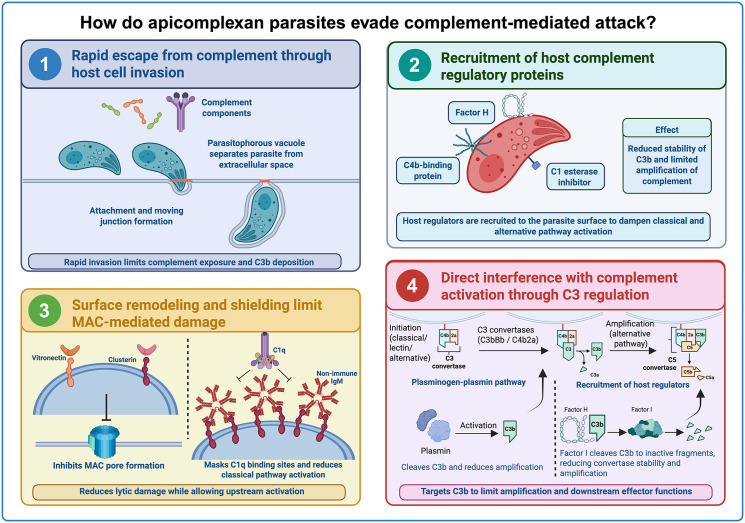
Complement evasion strategies in apicomplexan parasites. Apicomplexan parasites evade complement-mediated attack through four main mechanisms: (1) Rapid escape from complement through host cell invasion; (2) Recruitment of host complement regulatory proteins, such as factor H which binds C3b, C4b-binding protein that regulates classical pathway, and C1 esterase inhibitor, to reduce complement amplification; (3) surface remodeling and shielding, which interfere with MAC assembly and limit lytic damage, for example through the binding of host-derived factors or masking of activation sites, thereby reducing pore formation without fully blocking upstream complement activation; and (4) direct interference with complement activation at the level of C3, reducing amplification and downstream effector functions. Together, these strategies allow parasites to control complement activation while avoiding immune-mediated clearance. Whereas these strategies are well-characterized in selected species, comparable mechanisms in other apicomplexans remain incompletely defined and may vary depending on parasite biology and host cell context. Figure created in BioRender. https://BioRender.com/c1gs1vo under the agreement number: KR29NSQLYR.

*Toxoplasma gondii* recruits factor H and C4b-binding protein, reducing the stability of surface-bound C3b and attenuating amplification [18]. *Plasmodium falciparum* binds factor H via Pf92 and recruits C1 esterase inhibitor, thereby suppressing both alternative and classical pathway activation [19,20]. These interactions allow complement activation to begin but prevent its propagation (Fig 1).

Comparable mechanisms remain poorly defined in other apicomplexans. Available evidence is largely limited to functional observations of complement interaction, suggesting that similar outcomes may be achieved through alternative or yet uncharacterized mechanisms.

### (iii) Surface remodeling and shielding limit MAC-mediated damage

Mechanistic studies in *Plasmodium* indicate that complement-mediated lysis is limited through surface remodeling and recruitment of host-derived regulators that interfere with MAC assembly. These mechanisms act at the level of the terminal pathway by restricting insertion of C5b–7 complexes and subsequent pore formation [20].

*Plasmodium falciparum* recruits host proteins such as vitronectin and clusterin, which bind C5b–7 complexes and prevent their insertion into membranes. In addition, parasite surface proteins bind non-immune IgM in conformations that mask C1q binding sites, reducing activation of the classical pathway [4].

These mechanisms reduce lytic damage while allowing upstream complement activation to proceed. Similar strategies remain poorly defined in other apicomplexans and may vary according to parasite membrane organization and host cell context.

### (iv) Direct interference with complement activation through C3 regulation

Available mechanistic evidence indicates that apicomplexans interfere with complement activation by targeting C3, a key regulatory node in the cascade. These strategies do not block initiation; instead, they act on surface-bound C3b to disrupt amplification and downstream effector functions.

In *Plasmodium*, parasites recruit plasminogen and promote its conversion into plasmin, which cleaves C3b and reduces its capacity to sustain the amplification loop [19]. In parallel, recruitment of host regulatory proteins promotes factor I-mediated cleavage of C3b into inactive fragments, further limiting convertase stability (Fig 1). Together, these mechanisms regulate the deposition, processing, and inactivation of C3b on parasite surfaces, thereby limiting amplification and preventing progression to terminal complement activation. However, the specific parasite surface molecules that act as primary acceptors for covalent C3b deposition remain incompletely defined across most apicomplexans.

By altering C3b stability and turnover, these mechanisms reduce both amplification and progression to terminal pathway activity. Comparable molecular mechanisms remain largely unknown in other apicomplexans, although functional regulation at this step is likely conserved.

## Which proteins from each apicomplexan are involved in the modulation of the complement system?

While the mechanistic strategies that underlie complement evasion are increasingly well defined, the parasite-specific effectors that mediate these processes remain incompletely characterized across most apicomplexans. In contrast to other pathogens, where secreted or surface-exposed antigens directly target complement components, only a limited number of apicomplexan molecules have been functionally linked to complement modulation.

In *Plasmodium*, specific parasite ligands have been identified that provide a molecular basis for the complement evasion mechanisms described above. For example, parasite ligands such as Rh4 and PvEBP bind complement receptor 1 (CR1), facilitating immune evasion [21]. In addition, *P. falciparum* erythrocyte membrane protein 1 (PfEMP1) contains motifs within its heparin-binding domain that resemble host vitronectin, enabling interaction with terminal complement components and reducing MAC-mediated damage [8].

However, comparable parasite-derived effectors remain largely undefined in other apicomplexans, including *Toxoplasma*, *Eimeria*, and *Babesia*. Identifying these molecules will be essential to determine whether complement evasion relies on conserved effectors or represents a case of convergent evolution across the phylum.

## Concluding remarks

Apicomplexans evade complement by targeting central regulatory nodes without globally suppressing the system. Diverse mechanisms act on C3 activation and deposition, highlighting this step as a critical point at which multiple evasion strategies intersect.

These strategies are deployed differently across species, reflecting variation in extracellular exposure and host niches. While mechanistic understanding is concentrated in *Plasmodium* and *Toxoplasma*, evidence from other apicomplexans supports a broader role for complement across the phylum. Defining these conserved and divergent mechanisms will be essential for understanding host–pathogen interactions and for identifying strategies to enhance complement-mediated protection.
